# Unusual Onset of Hereditary Hemorrhagic Telangiectasia Due to Somatic Mutational Mosaicism: Case Report and Review of the Literature

**DOI:** 10.3390/children12121701

**Published:** 2025-12-17

**Authors:** Virginia Mirra, Margherita Rosa, Cristina Fontanella, Martina Mancuso, Fabio Antonelli, Alice Castaldo, Annalisa Allegorico, Maria Giovanna Russo, Mario Giordano, Alfonsina Tirozzi, Paolo Siani, Daniele De Brasi

**Affiliations:** 1 Chronic Diseases Hepatology and Nutrition Unit, “Santobono-Pausilipon” Children’s Hospital, 80129 Naples, Italy; 2 Department of Translational Medical Science, University of Naples Federico II, 80131 Naples, Italy; 3 Complex Structure of Pulmonology and UTSIR, AORN Santobono-Pausilipon, 80122 Naples, Italy; 4 Pediatric Cardiology Unit, University of Campania “Luigi Vanvitelli”, “Ospedali dei Colli”, 80131 Naples, Italy; 5 Medical Genetics Unit, Department of General and Emergency Pediatrics, AORN Santobono-Pausilipon, 80122 Naples, Italy

**Keywords:** telangiectasia, arteriovenous malformation, epistaxis, HHT, Rendu Osler

## Abstract

Hereditary Hemorrhagic Telangiectasia (HHT), also known as Rendu–Osler–Weber syndrome, is a disorder of angiogenesis characterized by mucocutaneous telangiectasias and visceral arteriovenous malformations. This rare autosomal dominant disorder is caused by pathogenic variants in the *ENG* and *ACVRL1* genes, and only 1–3% of case variants occur in *SMAD4*. HHT clinical manifestations include telangiectasias, epistaxis, and arteriovenous malformations in multiple organ systems. Clinical diagnosis is based on Curaçao Criteria. Here, we describe a pauci-symptomatic 10-year-old girl with an orbital and sinus infectious disease. Her clinical history was unremarkable, except for sporadic, self-limiting epistaxis episodes. She showed finger clubbing and low oxygen saturation levels on pulse oximetry, suggesting a chronic lung disease, and a large lung arteriovenous malformation. She also developed acute neurological symptoms, with evidence of multiple cerebral abscess lesions on MRI. HHT was therefore suspected and confirmed by genetic analysis, which revealed a de novo pathogenic variant in the *ENG* gene [c.1183G>T p.(Glu395Ter)] found in only 15% of the reads from NGS analysis, performed on peripheral blood lymphocytes, indicating a possible mutational mosaicism. This case outlines that HHT could present with unusual clinical symptoms highlighting the importance of diagnosis using both clinical criteria and genetic test.

## 1. Introduction

Hereditary Hemorrhagic Telangiectasia (HHT), also known as Rendu–Osler–Weber syndrome, is an inherited disorder of angiogenesis characterized by mucocutaneous telangiectasias affecting blood vessels and viscera, leading to abnormal vessel formation. It has a prevalence of 1:5000 and causes arteriovenous malformations (AVMs) in multiple organ systems, lacking intervening capillaries and resulting in direct connections between arteries and veins [1]. Common clinical manifestations include the following:

1. Telangiectasias, small, dilated blood vessels that can appear on the skin and mucous membranes, especially on the face, lips, tongue, and fingers (>90% of patients);
2. Epistaxis, frequent and sometimes severe nosebleeds beginning on average at 12 years of age, due to the presence of fragile blood vessels in the nasal mucosa (>90% of patients);
3. AVMs, which can occur in major organs such as lung, liver, brain and gastrointestinal tract. Depending on their location, AVMs can cause serious complications, such as stroke, brain hemorrhage, heart failure, or gastrointestinal bleeding. Pulmonary AVMs (PAVMs) (15–33% of patients) cause right-to-left shunting and predispose one to embolic stroke, brain abscess, arterial oxygen desaturation and other respiratory complications and may cause dyspnea and heart failure [1].

Other less common clinical signs are as follows:

4. Spinal AVMs, which may present with paralysis;
5. Vascular lesions in the pancreas (seen in 18% of individuals with HHT), though rarely causing clinical problems;
6. Pulmonary hypertension, resulting from systemic arteriovenous shunting in the liver and leading to increased cardiac output;
7. Mild-to-severe anemia, due to epistaxis, or less commonly GI bleeding, often requiring iron replacement therapy or, rarely, blood transfusion.

A diagnosis of HHT allows appropriate screening and preventive treatment for patients and affected family members [2,3]. The presence of three or more clinical manifestations, known as the Curaçao criteria, establishes the diagnosis. In symptomatic adults, the Curaçao criteria are routinely used to diagnose HHT. In adult patients with possible HHT (two of four criteria present), genetic testing is recommended to confirm diagnosis [4]. In children, skin and oral lesions are often absent until adulthood, and epistaxis may not manifest until adolescence [5]. Due to the age-dependent development of HHT symptoms, the Curaçao criteria have a low sensitivity for the diagnosis of HHT in pediatric age which represents a pitfall in the diagnostic process. Given the frequent lack of overt symptoms and signs, current guidelines recommend genetic testing be offered to asymptomatic children of a parent with HHT [4].

HHT represents a multisystemic disease, requiring multidisciplinary care, and its prognosis is closely related to the development of complications of AVMs in the lungs, liver and brain, and their early detection [6].

HHT is inherited in an autosomal dominant pattern. The condition is typically caused by pathogenic variants in the *ENG* (endoglin) or *ACVRL1* (ALK1) genes, both of which play a crucial role in the formation and maintenance of blood vessels. Pathogenic variants in endoglin (*ENG*, chromosome 9q34), activin A receptor type II-like 1 (*ACVRL1*/*ALK1*, chromosome 12q13), and *SMAD4* (chromosome 18q21) cause HHT1 (OMIM 187300), HHT2 (OMIM 600376), and combined Juvenile Polyposis/HHT (JP/HHT) syndrome (OMIM 175050), respectively [7,8,9].

A genotype/phenotype correlation is described in HHT. In fact, pulmonary and cerebral AVMs appear to be more common in individuals with *ENG* pathogenic variants, whereas hepatic AVMs appear more commonly in individuals with *ACVRL1* pathogenic variants. On the other hand, pulmonary hypertension in the absence of severe vascular shunting, a rare HHT manifestation, has occurred most commonly in individuals with pathogenic variants in *ACVRL1*, but it has also been reported in individuals with pathogenic variants in *ENG* [9].

## 2. Case Report

A previously healthy 10-year-old girl presented with profound, unexplained hypoxemia (oxygen saturation 79–83% on room air), digital clubbing, and central cyanosis, despite normal respiratory effort and a generally well appearance. These striking findings immediately suggested the presence of a significant right-to-left shunt and became the central clue guiding the diagnostic work-up toward PAVMs and HHT.

The patient had been referred for evaluation of right orbital cellulitis and pansinusitis, confirmed on facial Computed Tomography (CT). Her past medical history was otherwise unremarkable, aside from recurrent upper respiratory tract infections and rare, self-limiting epistaxis.

**Figure 1:**
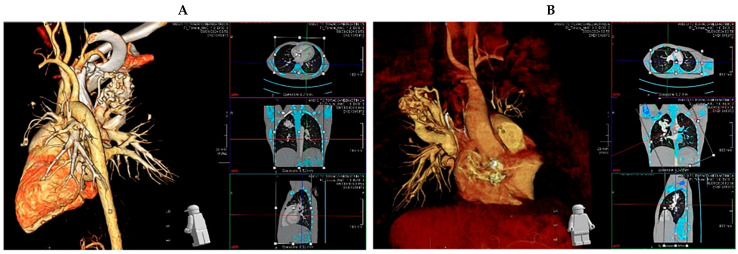
Frontal (**A**) and lateral (**B**) views from chest angio CT showing the largest AVM in the upper lobe of the right lung.

On examination, she exhibited right palpebral edema and paranasal sinus tenderness. In addition, digital clubbing, oral cyanosis, and decreased breath sounds at the right apex were noted. Pulse oximetry showed persistent saturations of 79–83% in room air, poorly responsive to supplemental oxygen and to 100% oxygen test. Arterial blood gas analysis confirmed marked hypoxemia (pH 7.44, pCO2 43.8 mmHg, pO2 29.8 mmHg, HCO3 29.7 Mmol/L). A color Doppler echocardiogram ruled out cardiac anomalies. Chest radiography revealed lobar opacities, and inflammatory markers were elevated, prompting initiation of intravenous ceftriaxone. Given the presence of cyanosis and oxygen desaturation with poor response to supplemental oxygen, it was necessary to rule out congenital or acquired methemoglobinemia. This was accomplished by measuring MetHb levels, which were found to be within the normal range (0.3%, normal value 0–1.5%). Chest CT angiography demonstrated multiple pulmonary arteriovenous malformations, the largest in the right upper lobe, suspected to be responsible for the chronic hypoxemia and clubbing (Figure 1A,B). After initial improvement on antibiotics and transition to oral therapy, the patient developed acute neurological symptoms, including headache, central facial palsy, and right arm weakness.

**Figure 2:**
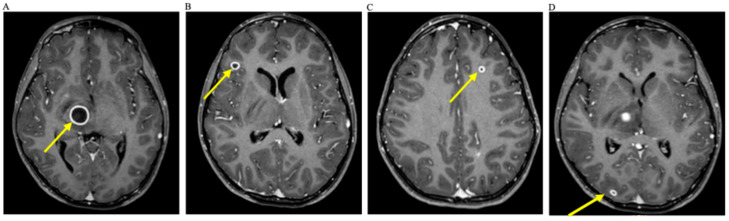
Brain localization of multiple abscesses on MRI at thalamus (**A**), right frontal lobe (**B**), left frontal lobe (**C**), and right occipital lobe (**D**), respectively (see arrows).

Brain MRI revealed multiple cerebral abscesses, the largest within the thalamus, and smaller lesions in the right occipital and frontal lobes, associated with vasogenic edema and mass effect (Figure 2). Additional incidental findings included multiple intracranial vascular anomalies—telangiectasias/capillary malformations and venous developmental anomalies.

**Figure 3:**
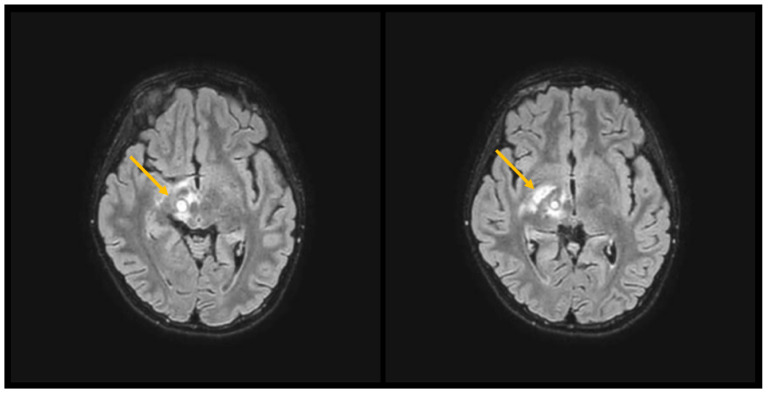
Brain MRI before (**left**) and after (**right**) 9 days of wide-spectrum antibiotic therapy: clear evidence of reduction in abscess dimension (see arrows).

Broad-spectrum intravenous antibiotic therapy with meropenem and linezolid was initiated and continued for eight and six weeks, respectively, with early clinical and radiological improvement; blood cultures remained negative. Amphotericin B was briefly added and discontinued after negative cerebrospinal fluid cultures for fungi and negative serum galactomannan and β-D-glucan. Corticosteroid therapy was administered in order to manage severe symptoms because of perifocal edema. Repeat MRI demonstrated substantial reduction in abscess size (Figure 3).

Angio MRI of the spine and abdominal and thoracic angio CT excluded additional AVMs. Based on the presence of two Curaçao criteria—recurrent epistaxis and multiple pulmonary AVMs—a clinical diagnosis of HHT was suspected.

Genetic testing was then performed, using a four-gene panel (*ACVRL1*, *ENG*, *GDF2*, *SMAD4*), associated with Rendu–Osler–Weber syndrome. Library preparation was performed using SureSelect Custom Constitutional Panel (Illumina Inc., San Diego, CA, USA), followed by sequencing on the Illumina NextSeq 500 platform [10]. A de novo variant in the *ENG* gene was identified, c.1183G>T p.(Glu395Ter), reported as pathogenic in the ClinVar database (accession number: RCV001262088.3), according to the ACMG criteria (PVS1, PP5, PM2), and associated with Rendu–Osler–Weber disease: notably, the variant was present in only 15% of the reads in the Next-Generation Sequencing (NGS) analysis, indicative of possible mosaicism.

Subsequently, Sanger sequencing was performed on epithelial cells from oral swabs to test for the *ENG* variant, confirming its presence in a different tissue. Genetic testing of parental blood samples for *ENG* variant was negative, suggesting a sporadic etiology of the genetic mutation.

**Figure 4:**
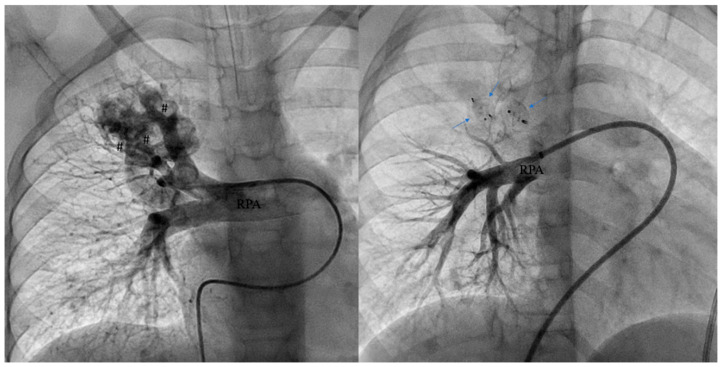
Pre-closure selective angiography of the right pulmonary artery (RPA) with a large PAVM of the upper lobe supplied by multiple vascular connections (#) (**left**) undergone trans-catheter embolization by three vascular plug (arrows) (**right**).

Subsequently, percutaneous PAVM embolization was performed to both avoid recurrent paradoxical embolization as well as achieve an adequate increase in arterial oxygen saturation. The PAVM was characterized by two major vascular connections (6 mm and 4 mm). The complex anatomy of PAVM required the implantation of three vascular plugs in three different segmental arteriolar connections: one Amplatzer Vascular Plug II 12 mm [Abbott, Plymouth, MN, USA] and one AmplatzerVascular Plug IV 5 mm [Abbott, Plymouth, MN, USA] to close the major connection (6 mm) and one Amplatzer Piccolo Duct Occluder 5–6 mm [Abbott, Plymouth, MN, USA] to close the other one (4 mm). The vascular plugs were preferred to coil to achieve a faster thrombosis and embolization of the vascular connections. The post-embolization pulmonary angiography highlighted a complete occlusion of the large PAVM with significant improvement in the arterial oxygen saturation (from 80 to 82% to 92–94%) (Figure 4). An angio CT scan is usually necessary one year after the procedure to detect the complete embolization of the PAVM and to exclude the formation of further vascular connections.

## 3. Discussion

Here we describe an unusual presentation of HHT in a 10-year-old patient with a medical history of only rare and mild epistaxis episodes, which resolved spontaneously, who was referred to our care because of orbital cellulitis and pansinusitis. Most pediatric patients with HHT are oligo- or asymptomatic and underdiagnosed [5]. In fact, even when they have AVMs causing right–left shunts, symptoms appear only when shunting exceeds 25% of total blood volume, with dyspnea, cyanosis, digital clubbing, and extracardiac murmurs [11,12]. The present patient maintained a normal quality of life with regular physical activity despite underlying respiratory issues, and although she was evaluated by a pediatric pulmonologist for recurrent respiratory infections, some mild clinical signs of chronic pneumopathy, namely finger clubbing and oral cyanosis, were not properly assessed. Thus, it is important for clinicians to pay attention to even minimal signs or symptoms of respiratory function impairment, when associated with even rare and mild episodes of epistaxis, and check for telangiectasias, anemia, other signs of bleeding, or a family history of epistaxis, and/or stroke or hemorrhagic events. Furthermore, a suspicion of PAVM and thus of HHT should be considered.

In our diagnostic work-up, CT pulmonary angiography was the key investigation for reaching the diagnosis of PAVM. Regarding other complementary investigations, the standard color Doppler echocardiogram was unremarkable and, in agreement with the vascular surgeon, a contrast (bubble) echocardiogram was not considered essential in this case because the presence of a pulmonary shunt, clearly documented through chest CT, was considered the primary cause of the oxygen desaturation and central cyanosis, making an unrecognized intracardiac shunt unlikely.

In the patient described here, a diagnosis of HHT was suspected based on two out of four Curaçao criteria (namely history of epistaxis, even if rare and mild, and lung arteriovenous malformations), in the absence of family history, confirming, in accordance with the literature data, the low sensitivity of the Curaçao criteria in children, when some signs and symptoms may not yet be evident. Further studies on diagnostic accuracy in pediatric age are needed.

During hospitalization, we encountered a serious and potentially life-threatening complication of PAVM: brain abscess. In fact, our patient exhibited acute neurological signs during hospitalization, which led to urgent neuroimaging, with a subsequent diagnosis of brain abscesses. The pathophysiology behind the frequent formation of cerebral abscesses in patients with PAVM is thought to be multifactorial and includes the absence of capillary filtration for bacteria due to the right-to-left shunts giving rise to venous emboli that can reach the peripheral circulation, together with a favorable environment for infection due to chronic hypoxia and polycythemia [11,12]. Thus, in case of a patient diagnosed with cerebral abscesses without other pathophysiological explanation, it is important to investigate for the presence of a PAVM [11,12,13,14]. In the present case, the absence of microbiological data from the cerebral abscesses represented a significant limitation in terms of both the diagnostic process and the management of the patient. While brain abscesses are usually polymicrobial, microbiological culture remains the gold standard for identifying the responsible pathogen. This identification is crucial for tailoring the antibiotic therapy to the specific infectious agent, potentially improving patient outcomes by ensuring more effective treatment and reducing unnecessary antibiotic exposure. In the absence of microbiological findings, as in our case, prompt broad-spectrum antibiotic therapy for both aerobic and anaerobic organisms is recommended, according to current guidelines. In particular, we chose the combination of meropenem and linezolid, considering that the patient, who had previously received intravenous ceftriaxone and oral amoxicillin–clavulanate and was in a symptomatic condition with severe radiological findings, warranted a second-line option. De-escalation criteria were followed in accordance with the guidelines and based on clinical and instrumental improvement [15].

From a genetic point of view, our patient presented with a pathogenic variant in the *ENG* gene and a clinical picture of PAVM, without brain, spine or liver AVMs. In fact, a genotype/phenotype correlation is described in HHT, as pulmonary and cerebral AVMs appear to be more common in individuals with *ENG* pathogenic variants, whereas hepatic AVMs appear more commonly in individuals with *ACVRL1* pathogenic variants, even though this correlation is not absolute [9]. The patient described here carried an *ENG* gene mutation and pulmonary AVMs, without hepatic AVMs, confirming the correlation between genotype and phenotype, and eventually directing clinical care and management.

The identification of a pathogenic variant in the *ENG* gene (c.1183G>T; p.(Glu395Ter)) with a variant allele frequency (VAF) of 15% strongly suggests the presence of somatic mosaicism in the patient: the absence of the variant in parental blood and its low VAF in peripheral blood lymphocytes of the patient supports a de novo post-zygotic mutation, likely contributing to a mosaic distribution of the pathogenic variant. Somatic mutational mosaicism is an uncommonly described genetic mechanism in HHT. Mosaicism is defined as the presence of two or more cell lines with different genotypes in one individual cell, developing from a singular zygote. The literature review reveals five previous articles describing mosaicism in HHT, whose clinical and genetic findings are summarized in Table 1 [16,17,18,19,20]. These observations underscore the importance of considering mosaicism in HHT cases, especially in a/oligo-symptomatic parents who test negative for pathogenic variants in the known disease-associated genes [10].

As shown in Table 1, cases previously reported in the literature demonstrate that the VAF varies widely among symptomatic individuals, ranging from ~10% to 55% in blood, saliva, buccal cells, hair follicles, and other tissues. These results suggest that low-level mosaicism may have clinical relevance and cause typical HHT symptoms, such as AVMs, brain abscesses, epistaxis, telangiectasias, and hepatic involvement. The data also show that the choice of tissue for genetic testing has a strong influence on detection: while peripheral blood may show a low VAF or even negative results, testing alternative tissues such as oral swabs, buccal cells or hair bulbs can reveal mosaic variants. These insights emphasize the need for careful tissue selection and consideration of mosaicism in genetic counseling, particularly for apparently unaffected parents who may carry low-level variants.

Whereas germline heterozygous mutations in HHT genes are well-known to cause the systemic disease, increasing evidence supports the “second hit” model to explain some differently localized manifestations, whereby biallelic loss of function via a somatic mutation in addition to a germline one, can result in localized vascular lesions. In our patient, the low-level somatic mosaicism (15% VAF) may have contributed to the localized development of the PAVM, consistent with a tissue-specific manifestation of HHT. While we cannot directly demonstrate a second-hit mutation in the affected lung tissue, we hypothesize that a post-zygotic loss-of-function event in a subset of cells may have acted in combination with the mosaic variant to produce the clinically evident PAVM, explaining the focal nature of the lesion. Given the absence of a family history of HHT, and the clinical presentation of PAVMs, their identification appears highly relevant, and it reinforces the role of somatic *ENG* mutations in PAVM pathogenesis. While NGS allows estimation of VAF, Sanger sequencing confirms the presence qualitatively in the oral epithelial cells but does not allow precise quantification. Importantly, low VAF may have influenced the relatively late onset and moderate severity of the PAVM presentation, as only a fraction of cells carried the pathogenic variant, possibly delaying the development of clinically significant shunting and related symptoms. These observations also highlight a potential genotype–phenotype correlation in mosaic states, suggesting that lower VAF may modulate timing and severity of disease manifestations. This highlights the need to consider mosaicism when germline testing is inconclusive or when clinical signs are localized or asymmetrical. Future studies should further investigate the prevalence and tissue specificity of such somatic events, which may have implications for diagnosis, prognosis, and possible future targeted therapies [21].

A notable case described by Tørring et al. (2018) [16] involved a woman with clinical features of HHT who was found to carry a frameshift variant (c.704dupC) in *ENG* gene in mosaic form, while her daughter harbored the same variant at the germline level. Despite low variant allele frequency, the mother exhibited typical HHT manifestations, including AVMs and mucocutaneous telangiectasias, though with milder severity. Similarly, Best et al. (2011) [18] reported other cases of *ENG* and *ACVRL1* mosaicism with full clinical expression, supporting the idea that mosaic pathogenic variants can cause overt disease.

Overall, the literature review and Table 1 synthesis suggest that symptomatic mosaic individuals can present with a wide range of VAFs, that tissue-specific testing significantly affects detection, and that these findings are highly relevant for genetic counseling, both for risk assessment and for guiding testing strategies in family members.

## 4. Conclusions

The present case highlights the importance of accurate patient anamnesis and comprehensive clinical assessment. It also emphasizes the importance of early diagnosis of PAVM in HHT patients for prognostic purposes, given the risk of cerebral abscess formation. Furthermore, it outlines the possible role of somatic mosaicism in causing HHT phenotype. In addition, we strongly recommend early genetic characterization of patients with HHT to better direct them to specific multidisciplinary follow-up care.

## Figures and Tables

**Table 1:** Genetic characterization of reported cases of HHT from the literature due to somatic mosaic mutations.

Reference	Patient	Sex	Age at Examination	Clinical Phenotype	Genetic Variant
[16]	Pt1	F	38	Epistaxis in infancy, PAVMs, Telangiectasias	*ENG* (NM_001114753.1):c.704dupC; (p.Val236Glyfs*98), (VAF: 55%); de novo
	Pt2, (Pt1’s mother)	F	76	PAVMs, Brain abscesses in older age, Telangiectasias	Negative for Sanger sequencing on blood; mosaicism detected in oral swab, (VAF: 11%)
[17]	Pt1	F	NA	Epistaxis, PAVMs, Telangiectasias	*ENG* (NM_001114753.1):c.1311+1G>A, identified on peripheral blood by exome sequencing (VAF: 38%);
	Pt2, (Pt1’s mother)	F	NA	Epistaxis, PAVMs, Telangiectasias and hepatic arteriovenous (AV) shunts	Negative on blood and oral swab; mosaicism detected in hair bulb, (VAF: 33%)
[18]	Pt1	F	43	Pulmonary arterial hypertension (PAH), Telangiectasias, Hepatic lesions consistent with focal nodular hyperplasia	*ACVRL1* (NM_000020.3):c.1451G>A mutation was present at 10% in blood; (VAF: 10%), saliva (VAF: 15%), buccal cells (VAF:16%)
	Pt2	F	55	Epistaxis, Telangiectasias, gastrointestinal, pulmonary and cerebral AVMs	*ENG* (NM_001114753.3):c.591delG (VAF: 29.5%) on peripheral blood
[19]	Pt1	M	1.5 months	Liver shunts, respiratory distress and possible telangiectasis	*ENG* (NM_001114753.3):c.1347_1348insCT (the mutant sequence peaks are much lower than the normal sequence peaks (15:85).
	Pt2	F	46	Epistaxis, Telangiectasis and family history of HHT symptoms	*ENG* (NM_001114753.3):c.511C>T (hair follicle and buccal swab samples show different levels of mosaicism)
	Pt3	M	10	Epistaxis, Telangiectasis and PAVM	*ENG* (NM_001114753.3):c.1080_1083delGACA (heterozygous, inherithed from her father)
	Pt3 father’s	M	NA	Frequent nose bleeds	*ENG* (NM_001114753.3):c.1080_1083delGACA (5% of mosaicism detected in leucocytes)
[20]	Pt1	F	40	Liver AVMs and a splenic artery aneurysm, PAH	germline mosaicisms for both *ACVRL1* (NM_001077401.2):c.1388del (Quantitative analysis revealed that 40% of the lymphocytes expressed this variant.) and *ACVRL1* (NM_001077401.2):c.1390del (quantitative analysis revealed that 40% of the lymphocytes expressed this variant.)
	Pt2 (son of Pt1)	M	24	Typical symptoms of HHT with varying degrees according to age.	*ACVRL1* (NM_001077401.2):c.1388del (heterozygous)
	Pt3 (son of Pt1)	M	18		*ACVRL1* (NM_001077401.2):c.1390del (heterozygous)
	Pt4 (son of Pt1)	M	7		*ACVRL1* (NM_001077401.2):c.1390del (heterozygous)

## Data Availability

The original contributions presented in the study are included in the article, further inquiries can be directed to the corresponding author.
